# Acyl CoA binding protein (ACBP): an autophagy checkpoint that can be targeted for improving cancer immunosurveillance

**DOI:** 10.1080/2162402X.2024.2413200

**Published:** 2024-10-07

**Authors:** Léa Montégut, Isabelle Martins, Guido Kroemer

**Affiliations:** a Team Metabolism, Cancer and Immunity, Centre de Recherche des Cordeliers, INSERM U1138, Équipe Labellisée - Ligue Nationale contre le Cancer, Université Paris Cité, Sorbonne Université, Paris, France; b Team Metabolism, Cancer and Immunity, Metabolomics and Cell Biology Platforms, Gustave Roussy Cancer Campus, Villejuif, France; c Department of Biology, Institut du Cancer Paris CARPEM, Hôpital Européen Georges Pompidou, AP-HP, Paris, France

**Keywords:** Caloric restriction mimetics, chemotherapy, immune checkpoint inhibitor, immunotherapy mammary carcinoma, non-small cell lung cancer

## Abstract

Acyl CoA binding protein (ACBP) encoded by *DBI* is a tissue hormone that limits autophagy in multiple cell types, hence acting as an extracellular autophagy checkpoint. We recently reported in *Molecular Cancer* that monoclonal antibodies neutralizing ACBP improve immunosurveillance of breast and lung carcinomas. Moreover, ACBP neutralization improves the outcome of neoadjuvant chemoimmunotherapy with PD-1 blockade in preclinical models.

## Main text

The immunogenicity of cancer cells, i.e., their capacity to elicit immune responses, is dictated by their antigenicity, i.e., their immunopeptidome recognized by T lymphocytes, as well as by their adjuvanticity, i.e., their capacity to emit danger signals that alert innate immune effectors, in particular conventional type-1 dendritic cells (cDC1).^1^ One of the intracellular processes that enhances adjuvanticity is autophagy, which favors the lysosomal secretion of adenosine triphosphate (ATP). Extracellular ATP acts on purinergic receptors to favor the chemotaxis of dendritic cells as well as their activation in the tumor bed.^2^

Based on this consideration, various methods for autophagy induction have been evaluated to enhance anticancer immune responses elicited by chemotherapy and chemoimmunotherapy involving PD1 blockade in mouse models.^3^ These methods initially included short-term fasting for 24 or 48 h as well as the administration of nontoxic autophagy inducing small molecules that we refer to as ‘caloric restriction mimetics’ (CRMs) such as hydroxycitrate, resveratrol and spermidine.^4^ We found that both fasting and CRMs improved the outcome of chemotherapy through effects that depended on the induction of autophagy in cancer cells, the release of ATP into the tumor microenvironment, as well as on the contribution of cDC1 and cytotoxic T lymphocytes.^4^ Encouraged by these results, mechanistic studies and drug libraries screenings led to identify aspirin, 3,4-dimethoxychalcone and isobacachalcone – among others – as CRMs. All these small molecules were endowed with the capacity to enhance chemo- or chemoimmunotherapy outcome.^4,5^ These cancer therapy-enhancing effects do not seem to depend on the proximal mode of action of each CRMs, which is rather heterogeneous, involving inhibition of protein kinase B (PKB/AKT targeted by isobacachalcone), the acetyltransferase E1A binding protein P300 (EP300, as reported for aspirin and spermidine), and insulin-like growth factor receptor (IGFR1), as well as activation of eIF4A hypusination (shown for spermidine), the deacetylase sirtuin-1 (shown for resveratrol) and transcription factor EB (TFEB activated by 3,4-dimethoxychalcone).^4,6,7^ Hence, irrespective of their precise mode action, autophagy enhancers can boost anticancer immune responses.

Recently, we developed a novel strategy for autophagy stimulation that does not rely on the use of small molecules. Indeed, we found that monoclonal antibodies targeting the extracellular pool of acyl CoA binding protein (ACBP) encoded by diazepam binding inhibitor (*DBI*) can induce autophagy in mice.^8^ ACBP is a phylogenetically conserved protein that possesses orthologues in fungi, plants and animals.^9^ ACBP is expressed in a close-to-ubiquitous fashion by multiple mammalian cell types.^8^ In human populations, ACBP can be detected in the plasma as a tissue hormone that increases with age, body mass index, as well as with signs of metabolic syndrome and systemic inflammation.^9^ Of note, in apparently healthy individuals, high plasma ACBP plasma concentrations constitute a risk factor for future cardiovascular events and cancer diagnosis. This effect is independent from age and BMI.^9,10^ These epidemiological associations suggest a causal involvement of ACBP in the aging process, as well as in major age-associated diseases.

Accordingly, knockout or knockdown of ACBP orthologs increases the lifespan of yeast and nematodes.^9^ Moreover, inhibition of ACBP by injection of a neutralizing monoclonal antibody (mAb) reduced myocardial infarction due to ligation of the left coronary artery and prevented accelerated cardiac aging induced by anthracyclines in mice.^9^ Similarly, ACBP neutralization reduced the progression of DNA damage-induced breast cancers and the incidence of urethane-induced non-small cell lung cancer (NSCLC) in preclinical experiments.^10^ Moreover, ACBP inhibition slowed down the progression of NSCLC induced by intravenous injection of TC1 cells, which seed into the lung. This latter effect was observed in immunocompetent mice but was lost in athymic mice lacking T cells,^10^ indicating that it relied on an intact immune system. Accordingly, ACBP inhibition enhanced the capacity of CD4 and CD8 T lymphocytes to mount interferon-γ responses against a model tumor antigen.^10^ Altogether, these results strongly suggest that the age-associated increase in circulating ACBP causatively contributes to the manifestation of cardiovascular and malignant diseases for which age is the most important risk factor.

**Figure 1.**
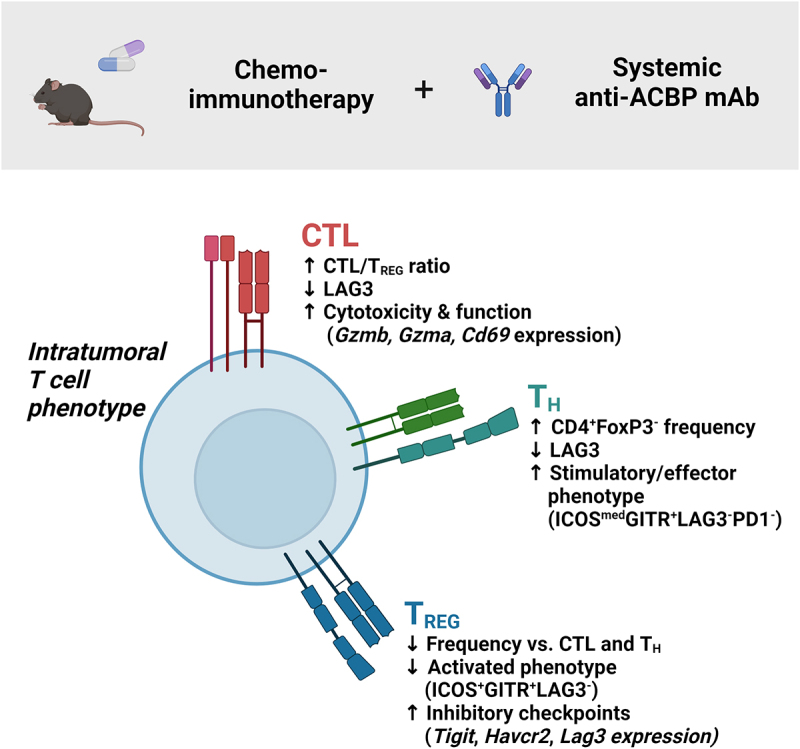
Improved T cell phenotype upon the combination of anti-acbp mAb with chemo-immunotherapy.

We also investigated whether anti-ACBP mAb would act similarly as other autophagy inducers (see above) and hence improve the outcome of chemoimmunotherapy. For this, immunocompetent mice bearing established subcutaneous MCA205 fibrosarcomas or TC1 NSCLC were treated with chemoimmunotherapy (oxaliplatin plus PD-1 blocking mAb) alone or in combination of anti-ACBP mAb. In both orthotopic cancer models, ACBP inhibition significantly amplified the effects of chemoimmunotherapy on tumor progression and animal survival.^10^ These effects were accompanied by favorable effects of anti-ACBP mAb on tumor-infiltrating T lymphocytes (Figure 1). Thus, in the context of chemoimmunotherapy, anti-ACBP mAb reduced the frequency of immunosuppressive regulatory T cells but expanded that of activated CD4 T cells in the tumor bed. In addition, in the CD8 T cell population, anti-ACBP mAb caused an increase in the expression of cytotoxic effector molecules but a reduction in the subset bearing exhaustion markers.^10^ These results plead in favor of an immunostimulatory effect of ACBP neutralization that is mediated by T lymphocytes. However, at this point it remains elusive whether these effects are direct, hence reflecting the effects of ACBP on T cells, or indirect on other cell types including malignant or myeloid cells.

Schematic summary of the phenotypic changes observed in the cytotoxic (CTL, CD3^+^CD8^+^), helper (T_H_, CD3^+^CD4^+^FoxP3^−^) and regulatory (T_REG_, CD3^+^CD4^+^FoxP3^+^) T lymphocytes compartments upon combination of anti-ACBP neutralizing monoclonal antibody during chemoimmunotherapy in the MCA205 skin sarcoma model.^10^ The reported changes were observed at the population and surface protein expression levels (flow cytometry data) and gene expression level (single cell RNA sequencing data). GITR: tumor necrosis factor receptor superfamily member 18; *Havcr2*: hepatitis A virus cellular receptor 2 gene; ICOS: inducible T cell costimulator; LAG3: lymphocyte-activation gene 3; PD1: programmed cell death 1; *Tigit*: T cell immunoreceptor with Ig and ITIM domains gene.

In conclusion, it appears that anti-ACBP mAb improves natural immunosurveillance against cancers and synergizes with chemoimmunotherapy. It is important to note that the effects of anti-ACBP mAb can be expected to be comparatively safe. Indeed, the knockout of the gene coding for ACBP in adult mice, as well as the induction of autoantibodies neutralizing ACBP over several months, have no discernible side effects.^8^ Future will tell whether the results that we obtained in mouse experiments can be safely translated to the clinics.
